# Water Meter Reading for Smart Grid Monitoring

**DOI:** 10.3390/s23010075

**Published:** 2022-12-21

**Authors:** Fabio Martinelli, Francesco Mercaldo, Antonella Santone

**Affiliations:** 1 Institute for Informatics and Telematics, National Research Council of Italy, 56124 Pisa, Italy; 2 Department of Medicine and Health Sciences “Vincenzo Tiberio”, University of Molise, 86100 Campobasso, Italy

**Keywords:** smart grid, smart meter, deep learning

## Abstract

Many tasks that require a large workforce are automated. In many areas of the world, the consumption of utilities, such as electricity, gas and water, is monitored by meters that need to be read by humans. The reading of such meters requires the presence of an employee or a representative of the utility provider. Automatic meter reading is crucial in the implementation of smart grids. For this reason, with the aim to boost the implementation of the smart grid paradigm, in this paper, we propose a method aimed to automatically read digits from a dial meter. In detail, the proposed method aims to localise the dial meter from an image, to detect the digits and to classify the digits. Deep learning is exploited, and, in particular, the YOLOv5s model is considered for the localisation of digits and for their recognition. An experimental real-world case study is presented to confirm the effectiveness of the proposed method for automatic digit localisation recognition from dial meters.

## 1. Introduction

A smart grid is defined as a self-sufficient system that allows the integration of any type and any scale generation sources to the grid to reduce the workforce targeting sustainable, reliable, safe and quality electricity to all consumers [1].

The rationale behind smart grid adoption is to allow an electricity network to be managed in an “intelligent” manner under various aspects or functions or by managing it efficiently for the distribution of electricity and for a more rational use of energy and, at the same time, minimizing any overloads and variations in the electrical voltage around the nominal value [2]. In a world that is increasingly attentive to energy consumption, waste and the use of environmental resources, smart grids are becoming essential. In fact, they are paired with another essential concept—namely, energy efficiency. With this term, we indicate the ability of a system, a network or a device to maximize the results for the same energy expenditure [3].

Such systems make it possible to open new perspectives for electricity companies, which, in addition to being able to proactively control the loads on the distribution network, have the opportunity to offer new services to their customers. Among these are, for example:

- the possibility of offering customized contracts for particular users, modifying the limit of the power supplied and applying more complex multi-hour tariffs or similar options;
- the opportunity to remotely disconnect some users from the network in the event of arrears or in the event of network overload, if provided for by contract;
- the possibility of having greater control over the distribution network in order to avoid fraud or abusive energy withdrawals;
- the opportunity to intervene automatically when there is a failure in the meter;
- the possibility of minimizing inefficiencies, due to the immediate acquisition of information.

All these opportunities allow for the optimization of the management of the network as a whole, thereby, not only providing better service to customers but also reducing a series of costs related to certain activities [4].

One of the fundamental components of smart grids is represented by the smart meter, which is aimed to obtain precise consumption data relating to electricity, creating a communication network between the various nodes of the smart grid, monitoring the incoming and outgoing energy flows for users with a production plant of renewable energy and providing two-way communication between consumers and services [5].

The principle behind smart meters is smart metering, which allows the implementation of energy efficiency strategies. In fact, the intelligent measurement and monitoring of consumption data are indispensable not only for energy distribution companies but also for end users who may become more aware and, consequently, act to improve their respective efficiency [6].

Thus, smart meters allow for obtaining precise consumption data relating to electricity, gas and running water. In the case of electricity, these devices create a communication network between the various nodes of a smart grid, monitoring the incoming and outgoing energy flows for users with a renewable energy production plant and optimizing the system with two-way communication in the case of a user connected to a digital energy community [7].

According to the “Power & Renewables” report by research firm Wood Mackenzie, between now and 2025, utilities will invest over $30 billion to install more than 300 million smart electricity meters. The installed devices will, thus, reach 1.3 million (https://www.woodmac.com/industry/power-and-renewables/, accessed on 21 November 2022).

In this context, the intelligent measurement and monitoring of consumption data are essential not only for service companies that distribute energy and gas but also for consumers to, thus, be more aware and, consequently, active in improving their efficiency [8]. Such monitoring, now that technology makes it smart and low-cost, becomes possible for end users as well [9].

The advantages of these systems are numerous for all parties involved:

- reduction of reading and contract management costs—operations that now become feasible remotely;
- higher reading frequency;
- network monitoring and maintenance optimization in case of leaks;
- possibility of free competition;
- user awareness of consumption and waste, given by the real-time measurement of consumption and related analysis due to efficiency algorithms;
- improvement of energy habits and increase in energy savings;
- reduction of energy costs for the user.

Despite this, there are still a huge number of non-smart meters in operation. We estimate that the Energy Company of Paraná (Copel), Brazil, takes more than 850,000 m readings per month [10].

According to Autorità di Regolazione per Energia Reti e Ambiente (https://www.arera.it/it/index.htm, accessed on 21 November 2022) (i.e., ARERA, the Italian Regulatory Authority for Energy, Networks and the Environment, which performs regulation and control activities in the sectors of electricity, natural gas, water services, the waste cycle and remote heat), a reading of the GAS meter is necessary after a number of time intervals, depending on the user contract:

- at least once a year for customers with consumption up to 500 standard per cubic metre (Smc)/year;
- at least twice a year for customers with consumption above 500 Smc/year and up to 1500 Smc/year;
- at least three times a year for customers with consumption above 1500 Smc/year and up to 5000 Smc/year;
- at least once a month for customers with consumption above 5000 cubic meters/year.

Similar considerations can be made for electricity and water meters. Starting from these considerations, in this paper, we propose a method based on deep learning for dial meter reading in the image under analysis that aims to (i) localise the dial meter, (ii) localise each digit into the meter and (iii) recognise the digits. The proposed method can be applied to several types of dial meter (for instance, for gas or electricity); however, in the experimental section, we focus on water dial meters by considering a dataset composed of more than 1500 water meter images.

The paper proceeds as follows: in the next section, the state of the art is discussed; in Section 3, we describe the proposed method for automatic dial meter reading; in Section 4, we present the experimental analysis results; and, finally, in the last section, our conclusions and future research plans to improve the proposed method are proposed.

## 2. Related Work

Gallo and colleagues [11] proposed a method that uses a Multilayer Perceptron (MLP) to locate the Region of Interest (ROI) of the meters (also denoted as counter region [11,12,13]), Maximally Stable Extremal Regions (MSER) to segment the digits, Histogram of Oriented Gradients (HOG) for feature extraction and Support Vector Machine (SVM) for digit recognition.

Nodari and Gallo [14] proposed a method named MultiNOD for gas cyclometer reading. It consists of a neural network tree with the sharing and resizing of features to perform counter detection and digit segmentation. The digit recognition stage was handled using Tesseract. This approach was later improved in [12], with the addition of Fourier analysis applied to the segmented image, in order to avoid false positives. Finally, SVM was employed for digit classification.

Tsai et al. [15] employed Single Shot MultiBox Detector (SSD) [16], a deep-learning object detector, to locate the counter region in energy meters. The authors reported an accuracy rate of 100% on their experiments, however, did not address the recognition stage.

Yang et al. [17] proposed a Fully Convolutional Sequence Recognition Network (FCSRN) for water meter analogue digit reading, with a novel loss function entitled Augmented Loss (AugLoss). AugLoss addresses the “middle-state” that can occur when the digit accumulator is changing from one display digit to the next one, usually outputting the old displayed digit. Their approach outperformed Recurrent Neural Networks (RNN) and attention-based models on the task of sequence recognition; however, the experiments were made in controlled images with cropped and aligned meters.

Gmez et al. [18] introduced a segmentation-free approach to perform meter reading. They trained a Convolutional Neural Network (CNN) to yield readings directly from the input images, without the need to detect the counter region. Although their approach has achieved promising results, the authors used a private dataset in the experiments and only compared their method with traditional algorithms that rely on handcrafted features, which are easily affected by noise and may not be robust to images acquired in adverse conditions [13].

Laroca et al. [13] designed a two-stage approach for AMR. The Fast-YOLOv2 model [19] was employed for counter detection, and three CNN-based models were evaluated in the counter recognition stage. The authors considerably improved their recognition results when balancing the training set in terms of digit classes through data augmentation techniques.

## 3. A Method for Automatic Dial Meter Reading

In this section, the proposed method for automatic digit dial meter reading is presented. In detail, we propose a method aimed to automatically read a meter from an image by exploiting deep-learning techniques. In particular, we focus on water meters; however, the method can be easily applied to other kinds of meters—for instance, electricity or gas ones.

**Figure 1:**
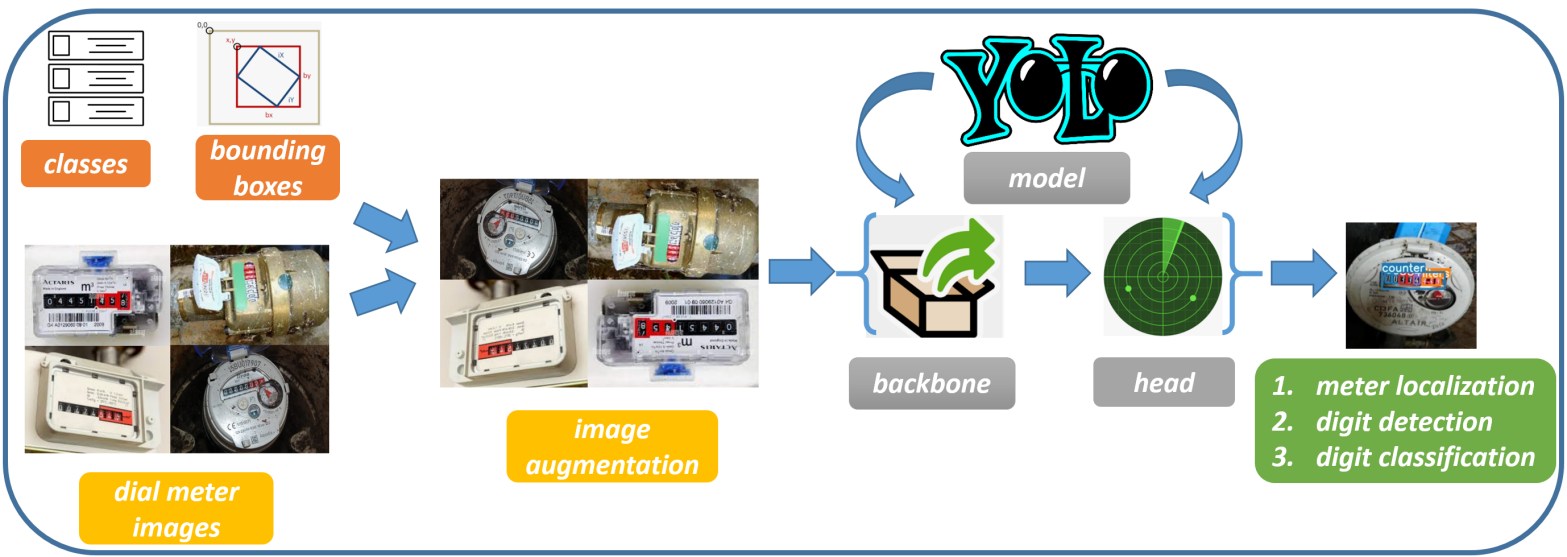
The workflow of the proposed method for automatic dial meter reading from images.

Figure 1 shows the workflow of the proposed approach, aimed to read the exact consumption of water in cubic meters.

To build an effective deep-learning model aimed to detect water consumption from water meters, a dataset composed of images with water meters is needed.

**Figure 2:**
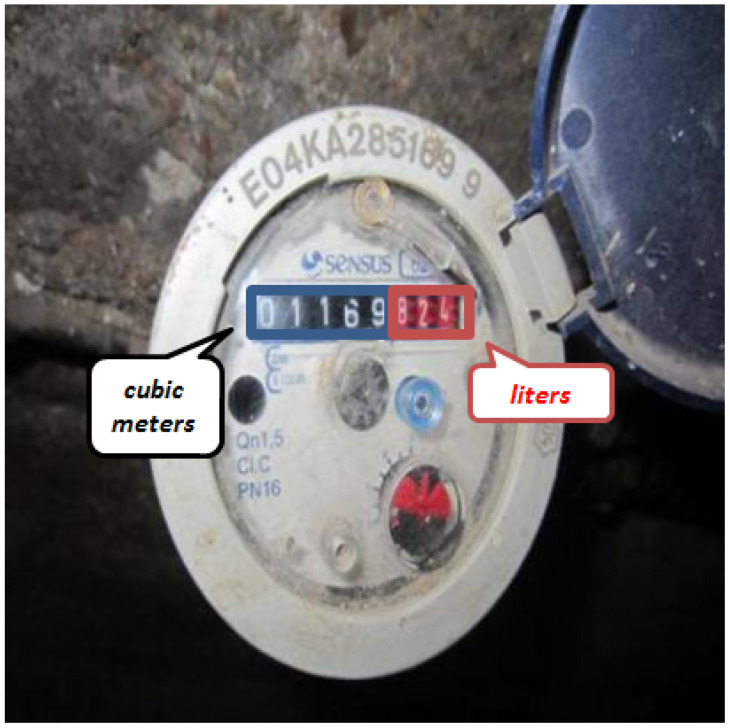
An example of a dial meter with details for the cubic meter quantity (in the black square) and the consumed water litres (in the red square).

Figure 2 shows an example of an image related to a water dial meter: the cubic meters are written in white on a black background, and, on a red background are the water litres (written in white).

Typically, water meters show two types of information:

1. The cubic meters of consumption with the digits in white on a black background or the reverse. This is the information that we need to extract from the water meter images.
2. The litres of consumption, with the digits in white on a red background or the reverse. This part of the meter will be ignored for the reading of the index as only the cubic meters are useful when billing consumers.

To build a model efficiently that is also able to correctly predict unseen images, images should be taken from different angles in different conditions and should belong to different water meter models. All of the images should have different sizes; however, after the images are obtained, we need to perform a preprocessing step aimed to resize the images to the same dimensions.

Once the images are obtained, we need to define the class for the detection of the bounding box for the meters and the digits: in total, we defined 12 classes of objects that we are interested in detecting in the water meter images, i.e., the 10 digits from 0 to 9, the part of the meter corresponding to the litres and the whole counter.

We labelled each picture by drawing the bounding boxes of each object found: this operation was performed by exploiting the Labelbox web application (https://labelbox.com/, accessed on 21 November 2022), i.e., a platform aimed to perform data annotation tasks.

In this way, the proposed model will be able to detect the area of the water meter related to the cubic meters, the area of the water meter related to the consumed litres and the position and the value of each single digit related to the cubic meters.

The next step is the *image augmentation*, i.e., is a set of techniques that expand the available dataset without actually collecting new elements: data augmentation applies controlled random changes to existing images, creating modified copies. It is used for automatic learning in artificial neural networks, which “learn” increasingly precisely as the available training dataset increases.

In particular, we apply data augmentation to generate dial meter images with controlled random changes, such as rotations, flips, cuts and trims [20]. The idea behind the data augmentation application in this case is to make the model suitable for performing effective reconnaissance regardless of the position in the image where the meter is present. Moreover, augmented data are used to solve the problem of overfitting of the statistical model to the observed data sample, which occurs when the model has too many parameters compared to the number of observations performed. Structured to recognize recurring patterns starting from the proposed data, the artificial neural network “learns” what it sees; however, it cannot find a generalized rule, and thus it easily mistakes patterns that are not yet viewed.

Once we have obtained the (augmented) dial meter images and the related details about the classes and the bounding boxes, we need a deep-learning model.

### The YOLO Model

In this paper, we resort to the “you only look once” (i.e., YOLO) model.

The YOLO [21] was proposed by J. Redmon et al. in 2016: it represents the first one-stage deep-learning detector. YOLO is an object-detection model, it is aimed to classify images and to detect their correct positioning within them.

The greatest difference from other networks is that YOLO considers a pipeline to perform the whole process in a completely independent way: the whole process of YOLO is: an image is taken as input, and we finally obtain two objects, a bounding box vector associated with each cell and the prediction of the class made for the object. Each image under analysis is divided into a matrix of SxS cells, where one cell is responsible for the object if it falls in the centre of the cell itself. The bounding box prediction has five components: (x, y, w, h and confidence). The coordinates (x and y) represent the centre of the bounding box with respect to the position of the cell of the grill. These coordinates are normalized to be between 0 and 1. The dimensions of the box (w and h) are also normalized to [0, 1], relative to the dimension image. In total, there are S × S × B * 5 outputs related to the predictions of the bounding boxes [22].

Compared to pre-existing object-detection models, YOLO is significantly faster, as demonstrated in [23,24]. This is mainly possible due to the fact that YOLO does not divide the recognition into several phases but predicts the bounding boxes, probability and classes of objects present in the input image in a single phase.

The reason why we resort to this model considering that we are aware that YOLO makes more localization errors [25], is that, compared to other object-detection deep-learning models, YOLO is less likely to recognize false-positives in the background of the image and is significantly faster [22,26]: this is the reason why YOLO is considered one of the best convolutional neural network models for object detection. There are several versions of the YOLO model. In this paper, we implement the model with a PyTorch (https://pytorch.org/, accessed on 21 November 2022) version of YOLOv5s (https://github.com/ultralytics/yolov5, accessed on 21 November 2022).

The architecture of YOLOv5s is shown in Listing 1.

**Listing 1.** The architecture of the YOLOv5s model for object detection.# *YOLOv5 backbone*backbone:  # *[from, number, module, args]*  [[−1, 1, Focus, [64, 3]],   [−1, 1, Conv, [128, 3, 2]],   [−1, 3, BottleneckCSP, [128]],   [−1, 1, Conv, [256, 3, 2]],   [−1, 9, BottleneckCSP, [256]],   [−1, 1, Conv, [512, 3, 2]],   [−1, 9, BottleneckCSP, [512]],   [−1, 1, Conv, [1024, 3, 2]],   [−1, 1, SPP, [1024, [5, 9, 13]]],   [−1, 3, BottleneckCSP, [1024, False]],  ] # *YOLOv5 head*head:  [[−1, 1, Conv, [512, 1, 1]],   [−1, 1, nn.Upsample, [None, 2, ’nearest’]],   [[−1, 6], 1, Concat, [1]],   [−1, 3, BottleneckCSP, [512, False]],    [−1, 1, Conv, [256, 1, 1]],   [−1, 1, nn.Upsample, [None, 2, ’nearest’]],   [[−1, 4], 1, Concat, [1]],   [−1, 3, BottleneckCSP, [256, False]],    [−1, 1, Conv, [256, 3, 2]],   [[−1, 14], 1, Concat, [1]],   [−1, 3, BottleneckCSP, [512, False]],    [−1, 1, Conv, [512, 3, 2]],   [[−1, 10], 1, Concat, [1]],   [−1, 3, BottleneckCSP, [1024, False]],    [[17, 20, 23], 1, Detect, [nc, anchors]],  ]

As shown in Figure 1, the YOLO network consists of a backbone (whose layers are shown in Listing 1), i.e., a convolutional neural network that aggregates and forms image features at different granularities, and a head (whose layers are also shown in Listing 1), aimed to consume features from the neck and take box and class prediction steps. Between the backbone and the head, there is the neck, i.e., a series of layers to mix and combine image features to pass them forward to prediction.

Once the YOLO model is trained, it will be able to perform the following operations on unseen dial meter images:

1. water meter cubic localization;
2. water litre localization;
3. digit detection in the water meter;
4. digit classification.

## 4. Experimental Analysis

In this section, we present the results of the experimental analysis aimed to show the effectiveness of the proposed YOLO model for water dial meter reading.

### 4.1. The Dataset

A dataset composed of 1000 annotated RGB pictures of water meters is considered.

The quality of the water meter images is heterogeneous as can be expected given that water meters are usually located underground. The water meters share a common shape but are not all identical, and some can be rotated also due to the data augmentation. The index part consists of (up to) eight rotating wheels to display the digits of the index: five white on black digits for the cubic meters followed by three white on red (or red on white) digits for the litres. By construction, it can happen that the wheels are rotating precisely at the moment the picture is taken; however, the considered images are unambiguous.

The dataset that we exploited is freely available for research purposes and for result replicability (https://challengedata.ens.fr/challenges/30, accessed on 21 November 2022) and it is composed of 1000 different images. After application of the data augmentation, we obtained the final dataset composed of a total of 1573 water meter images (i.e., the data augmentation generated 573 augmented images). From these 1573 images, we considered 1418 images for training (in detail, 1263 for training and 155 for validation) and the remaining 155 images for testing.

We resized all the images to the dimensions of 416 × 416. Relating to the model parameters, a batch size equal to 16 was considered, and we set the epoch number equal to 500. We performed augmentation by exploiting the Roboflow web application (https://roboflow.com/, accessed on 21 November 2022) by randomly rotating the pictures 90° clockwise, 90° counterclockwise and upside down.

Roboflow is a platform that enables developers to manage their computer-vision projects with their data. It allows the integration of images with their annotations from Labelbox and to apply some transformations on the images.

### 4.2. The Results

**Figure 3:**
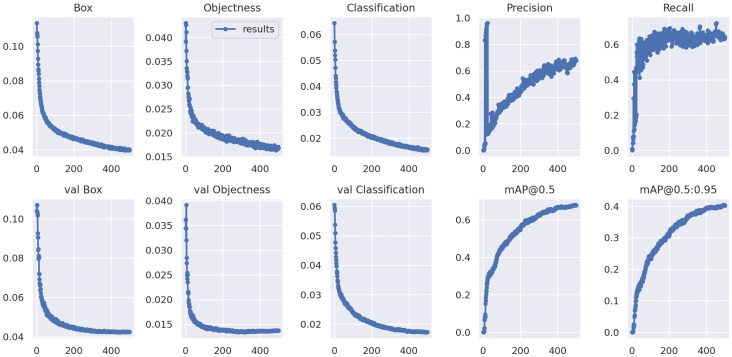
The results obtained from the experimental analysis.

Figure 3 is related to several plots aimed to show the performance of the proposed model for dial digit recognition.

In detail, Figure 3 shows 12 plots with 6 plots in each line. The first plot in Figure 3 (i.e., the *Box* plot) is related to the trend of the box loss metric. On the ordinates are the values of the loss, while, on the abscissa, the various epochs are represented. Generally, object detection involves localization and classification. Localizing multiple objects in an image is mainly performed using bounding boxes.

The bounding box is predicted with a loss function that gives the error between the predicted and ground truth bounding box: this is a loss that measures how “tight” the predicted bounding boxes are to the ground truth object and, considering that this is a loss function, the more it decreases, the more the network learns. This plot is related to the training step.

The second plot in Figure 3 (i.e., *Objectness*) is related to the objectness loss. Let us consider the objectness as the deep-learning model confidence that some object exists in a given box, and the class score is the conditional probability that there is an object in the box (i.e., the probability of class × the probability that an object exists in this box). The total confidence score for each class is, thus, the product of the objectness and the class score. Furthermore, in this case, the desirable behaviour is that, as the number of epochs increases, the objectiveness tends to zero. This plot is related to the training step.

The third plot in Figure 3 is the *Classification*. The classification is aimed to detect whether an object is present in the image and the class of the object. We consider, in this plot, a loss classification that measures the correctness of the classification of each predicted bounding box: each box may contain an object class or a “background”. This loss is usually called cross entropy loss. Cross entropy is used as the loss function for classification. This plot is related to the training step.

The *val Box*, the *val Objectness* and the *val Classification* plots are the loss trends, respectively, for the box loss metric, for the objectness and for the classification loss relating to the testing dataset: as in the previous plots, in these cases, we expect a decreasing trend when the number of epochs is increasing.

The fourth and the fifth plots (i.e., *precision* and *recall*) show the value for each epoch for the precision and recall metrics. Precision gives the proportion of positive predictions that are actually correct. It considers false positives, which are cases that were incorrectly flagged for inclusion. The *precision* can be computed as:

$$Precision=\frac{TP}{TP+FP}$$

Recall measures the proportion of actual positives that were predicted correctly. It considers false negatives, which are cases that should have been flagged for inclusion but were not. The *recall* can be computed as:

$$Recall=\frac{TP}{TP+FN}$$

Precision and recall should show an increasing trend as the number of epochs increase. In fact, this trend is shown in the graphs for both metrics; however, considering that precision and recall vary from 0 to 1, high performances are achieved. Both the recall and the precision are around the 0.7 value in the last epochs—this is indicative of what the deep-learning model has learned.

AP (average precision) is a popular metric in measuring the accuracy of object detectors, such as YOLO. The average precision computes the average precision value for recall values over 0 to 1. The calculation of the mean average precision (mAP) requires the intersection over union (IOU), precision, recall, precision–recall curve and AP. Object-detection models predict the bounding box and category of objects in an image. IOU is used to determine if the bounding box was correctly predicted.

The *IOU* indicates how much bounding boxes overlap. This ratio of overlap between the regions of two bounding boxes is 1.0 in the case of an exact match and 0.0 if there is no overlap. The *IOU* formula is shown below:

$$IOU=\frac{areaofoverlap}{areaofunion}$$

In the evaluation of object-detection models, it is necessary to define how much overlap of bounding boxes with respect to the ground truth data should be considered as successful recognition. For this purpose, IOUs are used, and mAP@0.5 is the accuracy when IOU = 50, i.e., if there is more than 50% overlap, the detection is considered successful. The larger the IOU, the more accurate the bounding box needs to be detected, and the more difficult it becomes. For example, the value of mAP@0.75 is lower than the value of mAP@0.5.

**Figure 4:**
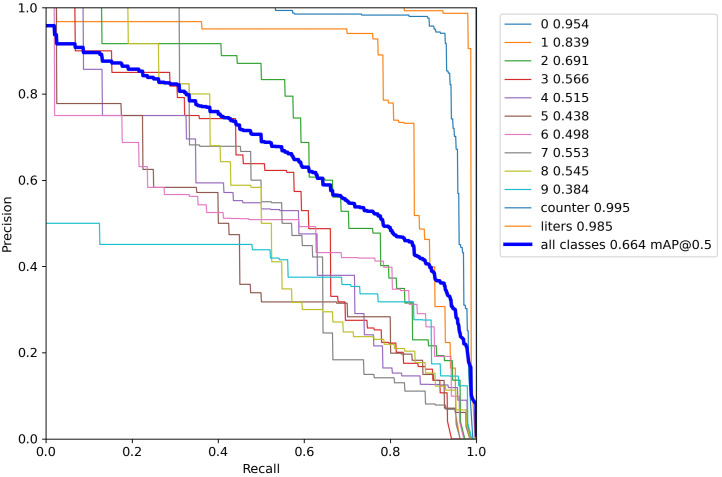
The precision–recall graph.

The mAP is an average of the AP values, which is a further average of the APs for all classes. Figure 3 shows, respectively, in the *mAP@0.5* and the *mAP@0.5:0.95* plots, the mAP value for IOU = 50 and IOU ranging from 50 and 95 (i.e., this value represents different IoU thresholds from 0.5 to 0.95, with a step size equal to 0.05) on the average mAP.). To discuss, in depth, the performance obtained in terms of the precision and recall, Figure 4 reports the precision and recall values on the precision–recall graph.

The trend of this plot is expected to be monotonically decreasing: in fact, there is always a trade-off between precision and recall. Increasing one will decrease the other. Sometimes the precision–recall graph is not always monotonically decreasing due to certain exceptions and/or lack of data; however, from the plot in Figure 4, we can see that this plot generally exhibits a decreasing trend. The precision–recall plot shows also the Area Under the Curve (AUC) values related to the 0–9 digit detection, the counter detection, the litre detection and the identification of all classes with *mAP@0.5*.

As previously stated, the precision–recall trend is expected to be monotonically decreasing: this behaviour is shown from the precision–recall plot related to all classes with *mAP@0.5* (with an AUC equal to 0.664). This value is the media of the AUC value of all considered classes: from the precision–recall graph in Figure 4, we can note that classes, such as the 0 and the 1, were able to obtain AUC values equal to 0.954 and 0.839, while the worst performances were obtained from the 4 and 5 classes with AUC values, respectively, equal to 0.515 and 0.438. Excellent performances were obtained from the counter class with an AUC equal to 0.995 and from the litre detection with a related AUC of 0.985.

**Figure 5:**
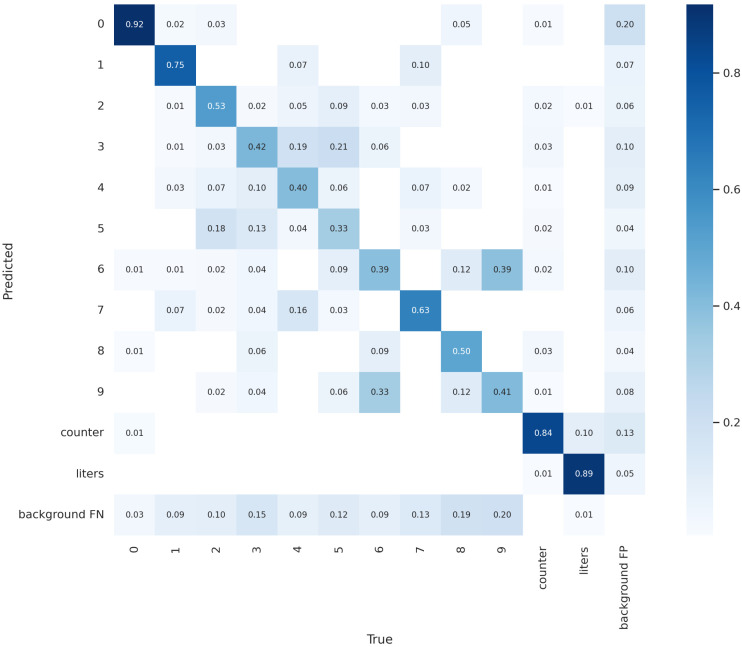
Normalised confusion matrix.

Figure 5 shows the normalised confusion matrix of the proposed YOLO model. We consider a confusion matrix in order to have a more precise idea of the classes obtaining the best and the worst performances. Moreover, with the confusion matrix, we can understand which instances are misclassified.

For instance, we confirmed that the model was able to correctly recognize the 0 digit: this was confirmed from the 0.92% value (i.e., 92 on 100 digits equal to 0 were recognised in the right class) shown in Figure 5. The 0.8% of misclassification are 0 digits erroneously classified by the model belonged to the 1 class (0.02%), 2 class (0.03%), 8 class (0.05%) and 9 class (0.01%).

Let us consider a class obtaining poor performance, for instance the 5-digit class. There were 0.33% of these instances correctly labelled as belonging to the 5 class. Another 0.18% of these instances were wrongly classified as belonging to the 2 class, 0.13% as the 3 class, 0.04% as the 4 class and 0.03% as the 7 class. No 5 digits were misclassified into the 0, 1, 6, 8 and 9 classes.

Relating to the counter detection, 0.84% of the counter were correctly localised. Similar performances were obtained for the litre localisation, where 0.89% of the litre counters were successfully recognised in the dial meter images under analysis.

### 4.3. Prediction Examples

**Figure 6:**
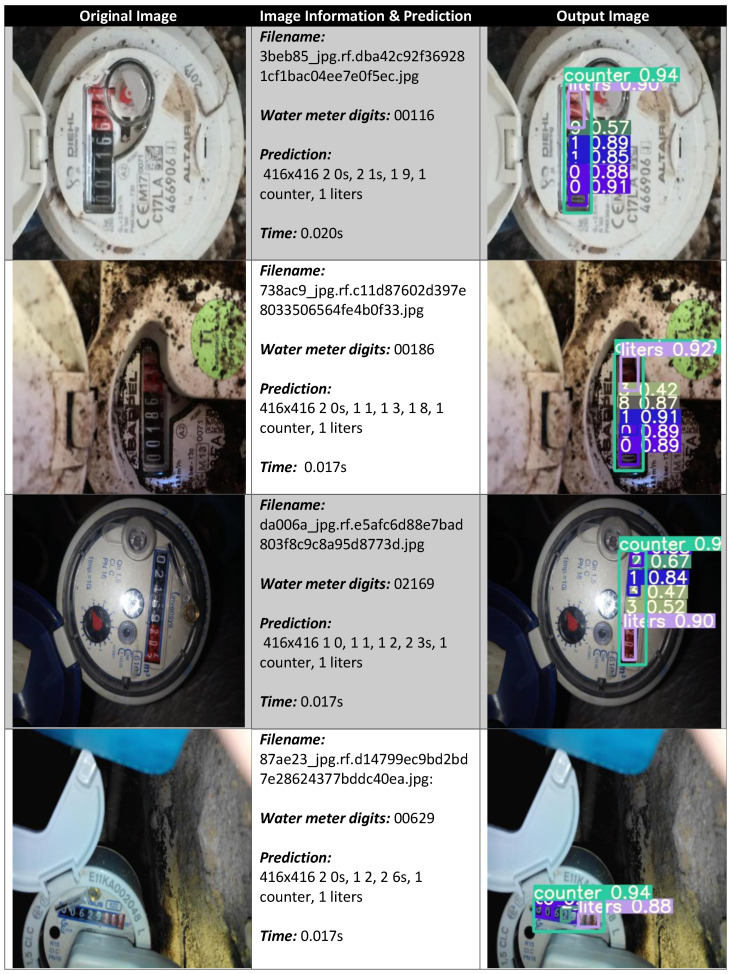
Four differentexamples of water meter detection performed by the proposed method: the first column shows the original image; the second column presents the water meter digits, the related prediction and the time employed for the detection; and the third column shows the image generated by the proposed method consisting of the overlaying of the original image with the details for the counter detection, the litre detection and the digit identification with the related detection percentage.

In order to show how the proposed method can be employed in the real-world, Figure 6 shows four examples of prediction performed by the proposed method.

In detail, in Figure 6, for each water meter image, we show the original image, the information about the image (i.e., the filename and the digits on the water meter), the prediction, the time to perform the prediction, and finally the image generated with the details for the counter and the litre highlighted as well as the prediction and the localisation for each counter digit with the details of the percentages for the digit predictions.

For instance, in the first image in Figure 6, the water meter counter shows the following digits: 00116, and the proposed method detected the following digits: two 0 s, two 1 s and one 9 in 0.020 s. From the image to the left, the counter was correctly localised with a percentage equal to 94%, the first two 0s with percentages equal to 91% and 88%, the two 1 s with percentages equal to 85% and 89% and the 9 with a percentage of 57% (this last percentage is low, and the digit in the water meter image is 6 and not 9: the model misclassified this last digit). In the left image, the litre counter was correctly localised with a percentage equal to 90%.

Regarding the second water meter, it is possible to note that, differently from the first one, it is in much worse condition, and the photo was taken in low light conditions. In the dataset, different images in different conditions and angulations were considered (due also to data augmentation) in order to increase the possibility to build a deep-learning model that is as generalised as possible. The water meter digits in the second images are 00186, and the proposed model predicted the following digits: 0 (with a percentage equal to 89%), 0 (with a percentage equal to 89%), 1 (with a percentage equal to 91%), 8 (with a percentage equal to 87%) and 3 (with a percentage equal to 42%).

Furthermore, in this case, we note only one digit misclassification, i.e., the model marked the 6 digit as a 3 and, similarly to the previous image, had a really low percentage: this is indicative that even the model was not confident of this prediction. The litre counter in this case was correctly localised with a percentage equal to 92% and, from the counter meter bounding box, we note that the localisation was correctly performed by the model. The required time for this prediction was 0.017 s.

The third image in Figure 6 exhibits an orientation opposite to the previous one. In this case, the digits on the water meter are 02169, and the proposed model detected one 0, one 1, one 2 and two 3 s. In this case, the model wrongly detected two 3 s instead of a 6 (with a percentage equal to 52%) and a 9 (with a percentage equal to 47%). The bounding boxes related to the counter and to the litres were correctly localised: the first one with a percentage equal to 90% and the second with a percentage of 90%. The time required to perform the detection was equal to 0.017 s.

In the last image shown in Figure 6, we note a water meter with a different orientation when compared with the previous three cases in order to demonstrate that the proposed method is generalizable to different kinds of images and water meter brands and is not dependant on the orientation, image luminosity and water meter distance. The digits in the water meter are 00629, and the proposed model detected two 0 s, one 2 and two 6 s: in this case, the misclassified digit was the 9, which was wrongly classified as a 6. Regarding the bounding boxes, the counter was correctly detected with a percentage of 94%, and the litre was also correctly detected with a percentage of 88%.

From an economic point of view, the proposed method can be easily implemented through an Arduino board that has a camera to take pictures of the dial meter, as well as an internet connection to send the readings. Alternatively, a smartphone would also be sufficient to take the photos and send the reading to a central server. The PyTorch library used for the implementation of the proposed method is available for the Android (https://pytorch.org/mobile/ios/, accessed on 21 November 2022) and Apple (https://pytorch.org/mobile/ios/, accessed on 21 November 2022) mobile operating systems; for this reason, the adoption of the proposed method is decidedly cheaper when compared to the adoption of smart meters.

## 5. Conclusions and Future Work

A smart grid is a combination of devices, computer programs and information-gathering systems that link together to quickly provide updated information and the status of an electrical system. A crucial and fundamental smart grid component is the smart counter, i.e., an electronic device (an evolution of the electric meter), which records the consumption of electricity. The replacement of all meters with intelligent devices that are able to send their own measurements autonomously is a long process, and meter readings are typically still performed manually by an operator at pre-established times. For these reasons, in this work, we proposed a method to make a dial meter capable of automatically reading the reported consumption.

The proposed approach relies on the adoption of the YOLOv5s deep-learning model for water meter reading. We performed experimental analysis with more than 1500 images, and we showed several case studies to demonstrate the effectiveness of the proposed method. The main problem that we encountered is the confusion between certain digits.

In future research, we will investigate the adoption of more augmentation techniques—for instance, blur, brightness and noise. Moreover, we plan to evaluate different YOLO architectures with the aim to obtain better performances by limiting digit misclassification. Different object-detection deep-learning architectures will be considered, including a transformer applied directly to sequences of image patches (as these have already been demonstrated to perform with good accuracy on object-detection tasks [27]) and applying the proposed method to video streams in order to also apply the proposed method to (low-cost) webcams for the remote transmission of measurements.
